# Potential Biomedical Applications of Collagen Filaments derived from the Marine Demosponges *Ircinia oros* (Schmidt, 1864) and *Sarcotragus foetidus* (Schmidt, 1862)

**DOI:** 10.3390/md19100563

**Published:** 2021-10-06

**Authors:** Marina Pozzolini, Eleonora Tassara, Andrea Dodero, Maila Castellano, Silvia Vicini, Sara Ferrando, Stefano Aicardi, Dario Cavallo, Marco Bertolino, Iaroslav Petrenko, Hermann Ehrlich, Marco Giovine

**Affiliations:** 1 Department of Earth, Environment and Life Sciences (DISTAV), University of Genova, Via Pastore 3, 16132 Genova, Italy; eleonora.tassara@edu.unige.it (E.T.); sara.ferrando@unige.it (S.F.); stefano.aicardi94@libero.it (S.A.); marco.bertolino@edu.unige.it (M.B.); mgiovine@unige.it (M.G.); 2 Department of Chemistry and Industrial Chemistry (DCCI), University of Genova, Via Dodecaneso 31, 16146 Genova, Italy; andrea.dodero@edu.unige.it (A.D.); maila@chimica.unige.it (M.C.); silvia.vicini@unige.it (S.V.); Dario.Cavallo@unige.it (D.C.); 3 Institute of Electronic and Sensor Materials, TU Bergakademie Freiberg, 09599 Freiberg, Germany; iaroslavpetrenko@gmail.com (I.P.); Hermann.Ehrlich@esm.tu-freiberg.de (H.E.); 4 Center for Advanced Technology, Adam Mickiewicz University, 61614 Poznan, Poland

**Keywords:** porifera, demosponges, biomaterial, collagen, spongin, wound healing

## Abstract

Collagen filaments derived from the two marine demosponges *Ircinia oros* and *Sarcotragus foetidus* were for the first time isolated, biochemically characterised and tested for their potential use in regenerative medicine. SDS-PAGE of isolated filaments revealed a main collagen subunit band of 130 kDa in both of the samples under study. DSC analysis on 2D membranes produced with collagenous sponge filaments showed higher thermal stability than commercial mammalian-derived collagen membranes. Dynamic mechanical and thermal analysis attested that the membranes obtained from filaments of *S. foetidus* were more resistant and stable at the rising temperature, compared to the ones derived from filaments of *I. oros*. Moreover, the former has higher stability in saline and in collagenase solutions and evident antioxidant activity. Conversely, their water binding capacity results were lower than that of membranes obtained from *I. oros*. Adhesion and proliferation tests using L929 fibroblasts and HaCaT keratinocytes resulted in a remarkable biocompatibility of both developed membrane models, and gene expression analysis showed an evident up-regulation of ECM-related genes. Finally, membranes from *I. oros* significantly increased type I collagen gene expression and its release in the culture medium. The findings here reported strongly suggest the biotechnological potential of these collagenous structures of poriferan origin as scaffolds for wound healing.

## 1. Introduction

Regenerative medicine currently needs innovative biomaterials characterised by low immunogenicity and toxicity and by good mechanical properties. Many biopolymers are available for their production, among them collagen—alone or combined with other ECM components, such as GAGs or elastin—is one of the most used and effective for these purposes [1]. Although skin and bones from bovine or porcine waste remain the primary source of this protein for regenerative medicine, the scientific community has recently shown a strong interest in marine collagen [2], consequently fish and various marine invertebrate collagens have been isolated and tested for tissue engineering applications [3,4,5]. Marine sponges in particular are one of the most promising sources among marine invertebrates for the production of collagen-derived biomaterials [6]. Marine sponge-derived collagen was tested for in vitro and in vivo studies, mainly for bone graft applications [7,8,9,10,11], and intact decellularized sponge 3D structures were used as bioinspired scaffolds for bone regeneration experiments [12]. Despite these several examples of the potential use of sponge collagen in regenerative medicine, there is only partial information on molecular characterisations of sponge-derived collagens [13,14], as well as on the molecular mechanisms involved in its biosynthesis [15,16].

Porifera is an extremely rich and biodiverse phylum, with more than 6,500 different species described to date [17]. These simple sessile animals are characterised by various shapes and textures supported by different structural solutions. Many species are characterised by a mineral skeleton made of silica or calcium carbonate, while others, commonly designated as keratose or horny sponges, have bodies formed exclusively by a flexible fibrous proteinaceous material commonly referred to as spongin [18,19]. The chemical-physical analyses of the abovementioned “spongin” lead back to a collagenic nature [18,20], but the co-presence of intercellular collagen fibres of smaller diameter in the same animals requires a better definition and characterisation of the various structures of collagen origin present in these sponges. Conventionally, the insoluble fibrous material isolated from the horny sponges after cell elimination by enzymatic treatment and centrifugation at low speed is designated as spongin B, while the collagenous suspension that can only be recovered through long and high-speed centrifugations is considered spongin A, or more generally, the sponges’ intercellular collagen fibres [18]. A third sponge collagenic material was furthermore found only in members of the *Irciniidae* family: peculiar collagen filaments intimately connected to the fibrous spongin matrix, forming a single extremely robust and flexible support unit, specifically described in the two genera, *Ircinia* and *Sarcotragus* [21,22]. Their function remains controversial. Previously, some studies have also advanced the hypothesis of structures acquired by parasites [23]. However, their detailed morphological and chemical-physical characterisation confirmed the collagenic nature of these structures [21], attesting to their function as a skeleton specialisation typical of these genera. Depending on the species, the size of these filaments varies from a few millimetres in length to a few microns, and, in most cases, they end with an ovoid knob. Their ultrastructural analyses show that they are composed of tightly connected sets of collagen fibrils, often containing iron hydroxide granules of Lepidocrocite (γ-FeO(OH)) [24], whose function remains unknown. Unlike spongin, these filaments are surrounded by a thin amorphous cuticle whose positivity to Alcian blue strongly suggest the presence of carbohydrate components. This overall organisation shapes the tight packaging of the collagen fibrils composing the filaments, and it confers them as a relevant resistance to enzymatic digestion [21]. Ultimately, these peculiar structural features of horny sponge filaments represent something unique of their kind, and in our opinion they could have in principle relevant characteristics as a raw material for the production of 3D composites [25], as well as new devices for regenerative medicine. 

The aim of this work is to develop a preliminary study to verify this hypothesis. For this purpose, two typologies of sponge-derived filaments are used: the large size ones from *Ircinia oros* (Schmidt 1864) and the small size ones from *Sarcotragus foetidus* (Schmidt 1862). 2D membranes were obtained by combining purified collagen filaments and intercellular collagen fibres from both sponge materials. Their ultrastructural, thermal and mechanical properties were analysed. Finally, their biocompatibility and ability to induce collagen and fibronectin production in fibroblast cell lines were tested to evaluate their potential as new biomaterials for skin regeneration in wound-dressing applications. 

## 2. Results and Discussion

### 2.1. Sponge Collagen Filaments (SCFs) Characterisation

#### 2.1.1. SCFs Microscopy Analysis 

**Figure 1:**
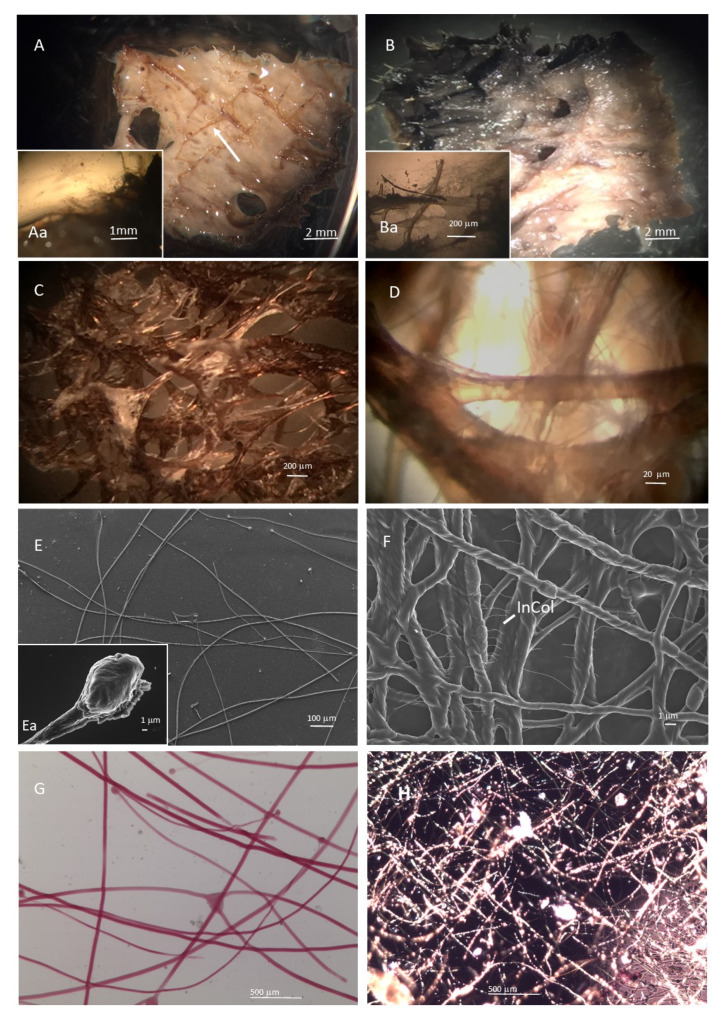
Morphology of collagen filaments derived from *I. oros* and *S. foetidus*. (**A**,**Aa**,**C**,**E**,**Ea**,**G**) Micrographs of collagen filaments isolated from *I. oros* tissues. (**B**,**Ba**,**D**,**F**,**H**) Collagen filaments isolated from *S. foetidus* tissues. (**A**,**Aa**,**B**,**Ba**) Stereo-microscopy analysis of fresh tissue. (**C**,**D**) Residual collagen filaments associated with spongin matrix at the end of the purification process. (**E**,**Ea**,**F**) Scanning electron microscopy analysis of isolated collagen filaments. (**G**,**H**) Micrograph of picro-sirius red staining of collagen filaments.

Microscopy analysis of fresh slices of *I. oros* and *S. foetidus* tissues showed their organic skeleton anatomy, articulated in a combination of main branched brown structures, commonly known as spongin, on which thin and clear filaments are tightly enveloped. With slight pressure on a free edge of the tissue, these filaments can be observed to emerge from the spongin’s main branch (Figure 1A,Aa,B,Ba).

Using a tissue dissociation enzymatic procedure combined with repeated extraction cycles in distilled water starting from 20 g (wet weight) of *I. oros* tissue, up to 400 mL of an aqueous suspension of collagen filaments isolated from the brown spongin matrix at a final concentration of 2 mg/mL could be obtained. The filaments extracted during the first two extraction rounds contained sediment residues and various sponge tissue debris and were thus discarded, while those obtained in subsequent cycles were progressively cleaner. After several extraction cycles, almost all the filaments have been removed from the brown matrix and collected in water suspension, and only a residual quantity still remained tightly knotted to the spongin structure (Figure 1C). Electron microscopy analysis of isolated filaments from *I. oros* confirmed the expected forms and dimensions, according to the descriptions previously published [21,26]. They showed a diameter of 13 μm, a length of several millimetres (up to 8 mm) and they ended with an oval knob (15–22 μm). The *I. oros* filaments were also strongly positive for picro-sirius red stain (Figure 1G), confirming their collagenous origin (Figure 1G).

A similar approach was followed to extract filaments from *S. foetidus*, with some specific adaptations. The filament dimensions, lower than the *I. oros* ones, and the high presence of inorganic sediments intimately distributed in the whole inner body of *S. foetidus* were the main cause of a greater difficulty in the isolation and cleaning procedure. Differently from the *I. oros* case, the reduced dimensions of *S. foetidus* filaments prevented the exploitation of a different speed of sedimentation to remove sediment residues and cell debris. Although the animal’s tissues are very rich in collagenic filaments, numerous repetitions of extraction cycles were necessary to obtain enough purified material; therefore, the first four cycles of extraction were discarded and only after the fifth cycle it was possible to obtain a filament suspension that is clean and pure enough. On the other hand, in these animals, the brown spongin structure intimately associated with filaments seemed to flake off more easily than in the previous case of *I. oros* (Figure 1D). Definitively, in *S. foetidus*, starting from 20 g of fresh tissue in our experimental conditions, it was possible to recover 200 mL of purified filament suspension with a final concentration of 2 mg/mL. Ultrastructural analyses showed filament diameters of 1–3 μm without any knobs at their ends. At a higher magnification, intercellular collagen fibres are clearly visible between the filaments, which are co-extracted during the purification steps (Figure 1F). Furthermore, *S. foetidus* filaments appeared intriguingly coated with an irregular sheath of inorganic material, formed by iron-containing compounds as shown by EDS analyses (Figure S1). This suggests that *S. foetidus* collagen filaments could contain iron-based mineral phases, as previously described in filaments of other sponges of the *Ircinia* genera [21]. The irregular distribution of these mineral sleeves found in our samples is partly a consequence of the extraction procedure, based on repeated cycles, as the ultrastructural analysis conducted on intact *S. foetidus* tissues shows that this coverage is considerably more homogeneous in origin (Figure S2). The remarkable discontinuous biomineral coatings prevent the homogeneous staining of this sample with picro-sirius red dye conversely to that obtained in *I. oros* filaments (Figure 1H). Notably, the presence of biogenic iron hydroxide intimately associated with collagenic material makes the biomaterials derived from this sponge species extremely interesting regarding biotechnological application, given the innumerable uses of composite collagen matrices with iron-based minerals [27,28,29].

#### 2.1.2. SCFs Biochemical Analysis 

##### Amino Acid Composition 

Table 1 shows the amino acid composition of hydrolysed SCFs derived from *I. oros* and *S. foetidus*, expressed as residues ‰. The amino acid profile of the collagen filaments of both sponges is similar. Therefore, this suggests that despite very different sizes, these filaments are formed by similar collagen molecules. Like rat and codfish collagen, sponge collagenous filaments have glycine as their major amino acid, as shown in 359/1000 and 385.46/1000 residues, in *I. oros* and *S. foetidus* filaments, respectively. Proline contents in collagen filaments from *I. oros* and *S. foetidus* resulted in 57.06 and 67.29 residues ‰, respectively. Together with glycine and proline, hydroxyproline and hydroxylisine are amino acid characterising collagens. In particular, the total amount of hydroxyproline residues in collagen can affect the thermal stability of collagen [30]; furthermore, its content is higher in rat collagen than sponge collagenous filaments, as it suits the higher mammalian body temperatures. Conversely, its content level is lower in codfish than in sponge collagenous filaments, due to the lower temperature in which this animal lives, compared to *I. oros* and *S. foetidus,* which typically grow in low depths of the Mediterranean Sea. As observed for proline residues, the lysine content in the collagenous filaments of sponges is also lower than in vertebrate collagens; however, its percentage of hydroxylation is higher. Highly hydroxylated collagen has been reported previously within skeletal structures of glass sponges [31]. Here, the total hydroxylysine content is remarkably higher in the sponges’ filaments than in vertebrate collagen, resulting in almost double compared to codfish collagen. Hydroxylysine residues are known to be involved in crosslink reactions during fibre assembling [32], and their peculiar abundance could explain the strong insolubility of filaments themselves. Another very interesting fact that emerged in this study is the high level of aspartic acid content in sponge filaments compared to vertebrate collagen. This last feature, already described in several other poriferan collagens [21,33,34,35], is again something peculiar in the context of sponge collagens, and it could explain their difficulty in being solubilised in acidic conditions, contrary to the collagens of higher organisms.

##### Amino Acids, Glycosaminoglycans (GAGs) and Iron Content

The results obtained by the quantitative analysis of total amino acids (GAGs) and iron is showed in Table 2. These data indicated that the proteinaceous component in *I. oros* filaments are double compared to *S. foetidus* filaments. Conversely, the GAGs amount obtained by Alcian blue quantitative assay were higher in *S. foetidus* filaments compared to *I. oros* counterpart, resulting in 28.49 ± 8.3 μg/mg and 12.28 ± 5.4 μg/mg, respectively. 

Finally, the quantitative evaluation of the iron component in SCFs obtained by inductively coupled plasma atomic emission (ICP–AES) confirmed the previously obtained data in *I. oros* samples [22], and provides us with a quantitative indication on the biomineral component identified in *S.foetidus* collagen filaments through EDS (Figure S1) in ultrastructural analyses.

##### Sodium Dodecyl Sulfate Poly Acrylamide Gel Electrophoresis (SDS-PAGE)

**Figure 2:**
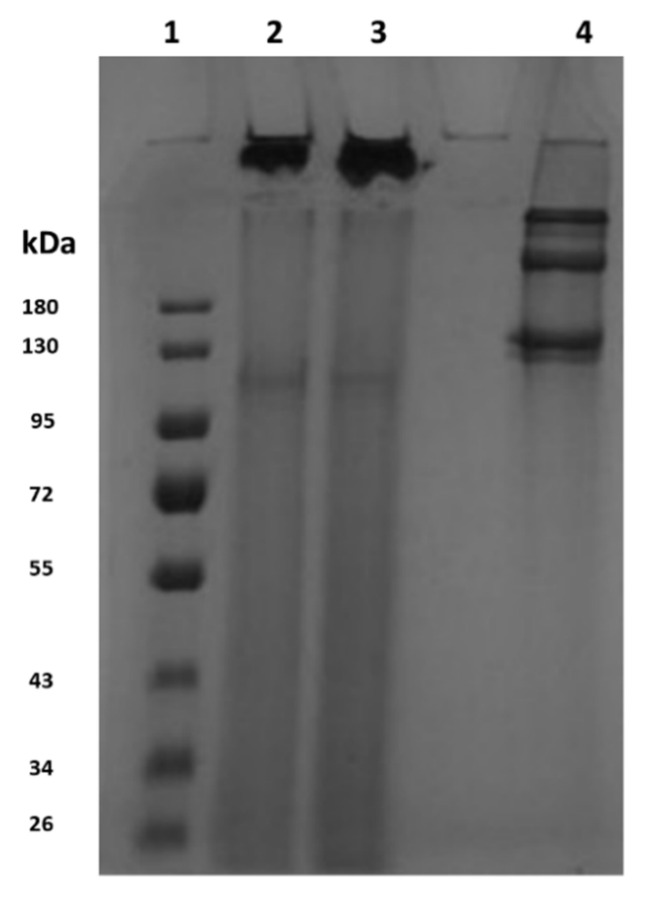
SDS-PAGE analysis of purified SCFs. A total of 40 μL of 2 mg/mL sponge filament suspensions crushed in acid-washed glass beads in 1× gel loading buffer and heated at 90 °C for 5 min were loaded in a 7% polyacrylamide gel. After electrophoresis, gel was stained with colloidal Coomassie blue staining as described in Section 4.3. Lane 1: standard molecular weight markers (kDa); Lane 2: *I. oros* collagen filaments; Lane 3: *S. foetidus* collagen filaments; Lane 4: rat tail type I collagen.

The electrophoretic profile of purified *SCFs* from *I. oros* and *S. foetidus* could be obtained only after their destruction by glass beads in 1× denaturing gel loading buffer (Figure 2). No differences in electrophoretic patterns between the sponge species were observed. In both samples, a weak band corresponding to 120 kDa, consistent with the typical size of the fibrillar collagens α-chains of higher animals [36] and other marine sponge species, was detected. In particular, a similar molecular size was observed in the SDS-PAGE analysis of fibrillar collagen extract purified from the marine sponge *Chondrosia reniformis* [37]. The presence of a single band suggests that the fibrillar collagens of these sponge filaments should be homotrimers forming homotypic fibres.

### 2.2. Sponge Collagen Filament Membranes (SCFMs) Characterisation

#### 2.2.1. SCFM Surface Morphologies 

**Figure 3:**
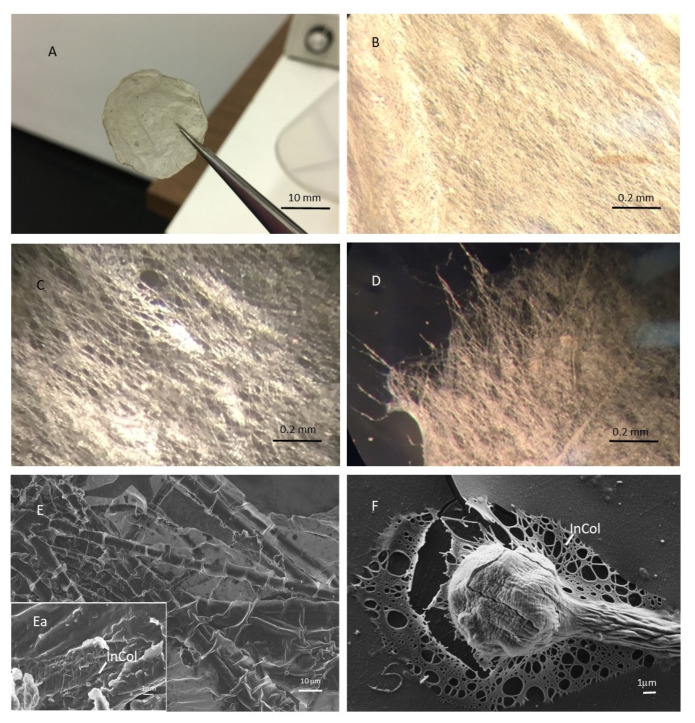
*I. oros*-derived membranes morphology. (**A**) Photograph of whole *I. oros*-derived membrane. (**B**,**C**) Micrographs showing the texture *I. oros*-derived membranes. (**D**) Micrograph showing the jagged edge of the *I. oros*-derived membrane. (**E**,**Ea**) Scanning electron microscopy analysis showing the texture of the *I. oros*-derived membrane. (**F**) Scanning electron microscopy analysis showing the filament knob embedded in intercellular collagen fibre matrix. **InCol**: intercellular collagen fibres.

**Figure 4:**
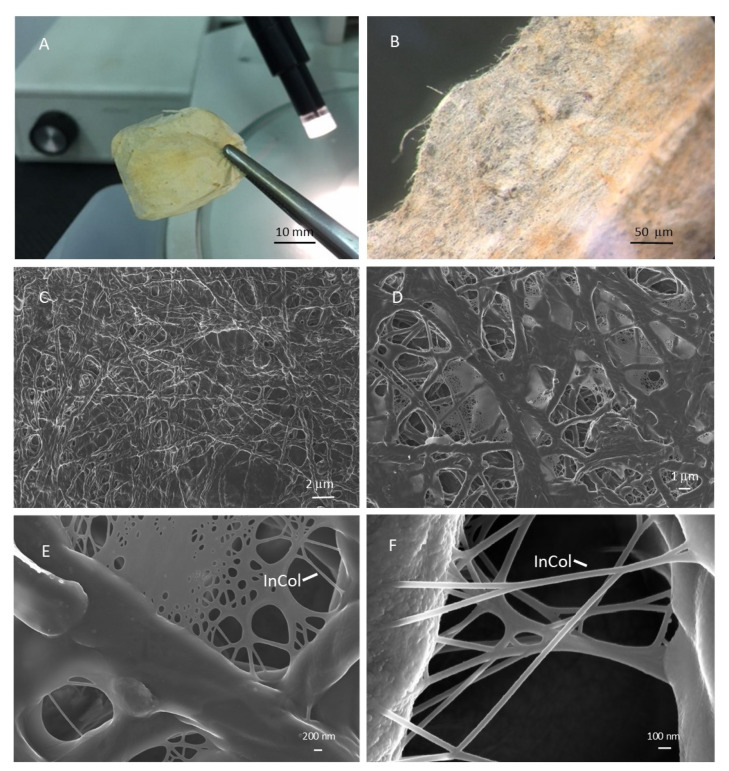
*S. foetidus*-derived membranes morphology. (**A**) Photograph of whole *S. foetidus*-derived membrane. (**B**) Micrographs showing the jagged edge of the *S. foetidus*-derived membrane. (**C**–**F**) Scanning electron microscopy analysis showing the texture of the *S. foetidus*-derived membranes. **InCol**: intercellular collagen fibres.

When SCFs derived from *I. oros* and *S. foetidus* were cast and dried in silicon mods, it was possible to recover thin, light membranes that were extremely smooth to the touch (Figure 3A and Figure 4A). The texture of *I. oros*-derived membranes was clearly visible under an optical microscope at low magnification, thanks to the larger size of its filaments (Figure 3B–D). The membrane structure appeared to be a disorganised web of filaments bound by a bright, transparent matrix. The pores delimited by the mesh of the membrane texture spanned an average value of 624.09 ± 291.49 μm^2^. Ultrastructural analysis showed that the *I. oros* collagenic filaments in the membranes were intimately associated with intercellular collagen fibres that were co-extracted with the filaments and that, once dried, formed a whole with the filaments themselves (Figure 3E,F). At light microscopy, the network of the membranes obtained from the collagen filaments of *S. foetidus* was more compact than that derived from *I. oros* filaments (Figure 4A), and their mesh was only visible in the edge through high magnification (Figure 4B). In electronic microscopy with high magnification, these membranes were formed by a dense weave of filaments bound by intercellular collagen fibres with a smaller diameter (Figure 4C–F). Here, the pores delimited by the mesh of the membrane texture spanned an average value of 8.90 ± 5.61 μm^2^. The remarkable differences in the filament networking and the pore texture can be justified by the different diameters of the filaments (13 μm for *I. oros* and 1–3 µm for *S. foetidus*), and by their distinct surface characteristics. *S foetidus* filaments, in particular, have a very peculiar coating of iron oxide, and this feature could play a role in the intercellular matrix protein-filament interactions in the membrane structure.

#### 2.2.2. Thermal Properties

**Figure 5:**
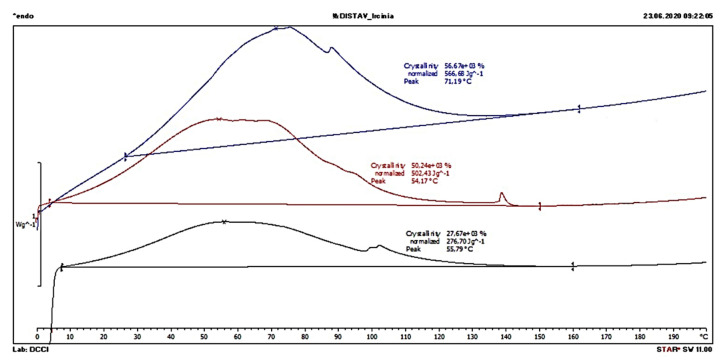
DSC analysis of SCFMs. Thermograms of 3 mg of dry *I. oros*-derived membrane (blue line), *S. foetidus*-derived membrane (black line) and commercial porcine collagen membrane Bio-Gide® (red line).

Differential scanning calorimetry (DSC) thermograms of dried SCFMs and of one commercial collagen membrane (BioGide^®^, chosen as comparison biomaterial) are shown in Figure 5. An endothermic peak, with a maximum temperature point (Tmax) of 71.19 and 55.79 °C, was observed for *I. oros*-derived and *S. foetidus*-derived membranes, respectively, while BioGide^®^ commercial collagen membranes showed a Tmax at 54.17 °C. Therefore, compared to BioGide^®^, both SCFMs showed higher thermal stability. However, while *S. foetidus*-derived membranes differed by a few degrees, those derived from *I. oros* filaments showed a higher difference compared to commercial collagen membranes. A high thermal stability of solubilised collagen molecules is related to the high level of proline/hydroxyproline [30]. However, a small difference in the amount of these amino acids was observed in the collagen filaments isolated from both species (Table 1). Furthermore, their content is considerably lower than in mammalian collagen, where commercial membranes are formed. It is therefore evident that other chemical characteristics must come into play to ensure the high thermal stability found in the *I. oros*-derived membranes. Typically, commercial collagen membranes were obtained from controlled in vitro fibrillogenesis of solubilised tropo-collagen. Conversely, SCFMs are obtained by casting and drying the collagen filaments formed by tightly assembled intact collagen fibres that, for *I. oros* filaments, are further enveloped in Alcian blue positive sugar sheath [21]. The thermal stability of a collagen-derived biomaterial is increased by its crosslink level [38]; hence, the high thermal stability observed in *I. oros*-derived membranes could be related to the interchain cross-links involved in the association of collagen fibres. *S. foetidus* filaments are partially covered by iron biomineral; however, the presence of mineral nanoparticles does not seem to affect the thermal stability of collagen-derived scaffolds [38,39]. This could explain the low Tmax difference between commercial collagen and *S. foetidus*-derived membranes. 

#### 2.2.3. Mechanical Properties

**Figure 6:**
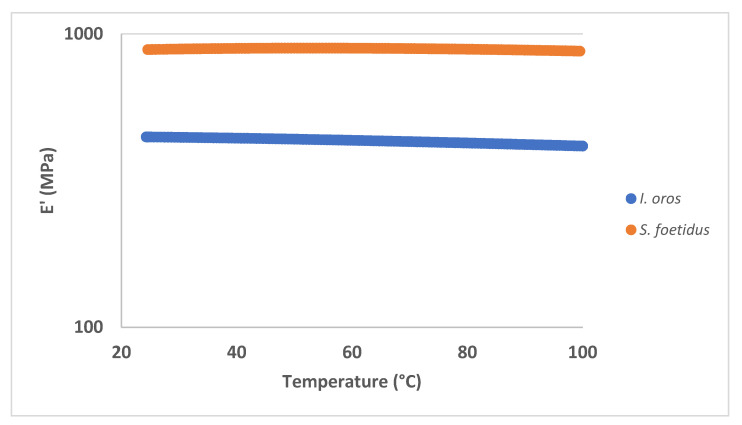
Mechanical test of SCFMs. DMTA spectra of *I. oros*-derived (blue curve) and *S. foetidus*-derived (orange curve) membranes measured in extensional configuration with a frequency of 1 Hz and an extensional stress of 0.1 MPa.

DMA and DMTA were carried out on the prepared SCFMs in order to compare their overall mechanical performances. As summarised in Table 3, *S. foetidus*-derived membranes present much higher elastic (E’) and loss modulus (E”), with respect to those derived from *I. oros* in the same experimental conditions. This greater mechanical stiffness may be ascribed to different reasons. First, the smaller size of the filaments’ diameter from which *S. foetidus*-derived membranes are formed generates a more compact texture with smaller diameter pores, which can in turn enhance the sample rigidity [40]. Second, despite that in both types of membranes the filaments are bound by intercellular collagen fibres co-extracted with the filaments, the smaller size of the *S. foetidus* filaments could allow these filaments to interact better with the intercellular collagen fibres to form a continuous structure, while this is harder in the *I. oros*-derived membranes due to the larger size difference between the filament diameter and dispersed intercellular collagen fibres. Finally, the iron biomineral coating present in *S. foetidus* filaments is likewise responsible for a further enhancement of the sample mechanical performances with respect to the *I. oros*-derived membranes [41]. Additionally, as shown in Figure 6, it is noteworthy that both the prepared SCFMs are characterised by a high thermal stability within the investigated temperature range. However, *S. foetidus*-derived membranes appear to once again perform better compared to the *I. oros*-derived ones, presenting a lower decrease in the elastic modulus as the temperature is increased.

Generally speaking, both SCFMs are characterised by suitable mechanical properties for a broad range of biomedical applications, ranging from scaffolds for tissue regeneration to wound-healing patches.

#### 2.2.4. In Vitro Degradation Evaluation 

**Figure 7:**
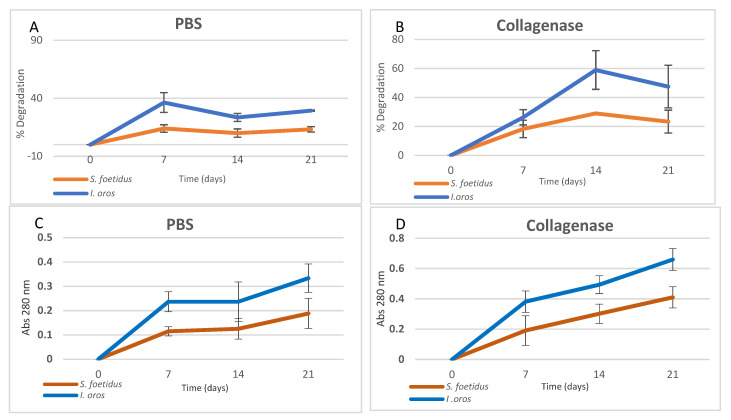
In vitro degradation test. Percentage degradation in PBS (**A**) and in 0.1 mg/mL collagenase solution (**B)** obtained as dry wight difference. Organic compound release from SCFMs in PBS (**C**) and in 0.1 mg/mL collagenase solution (**D)** obtained by 280 nm absorbance measurement of the incubation media. (*n* = 3, mean ± standard deviation).

The degradation rate of SCFMs in PBS or collagenase solution was first investigated by evaluating the percentage of weight loss as a function of the degradation time. The percentage weight loss of SCFMs within 21 days in PBS (pH 7.4) is shown in Figure 7A. For each membrane type, the main weight loss was detected within seven days, while insignificant weight loss was observed after 14 and 21 days. After seven days, *I. oros*-derived membranes exhibited a weight loss rate of 36.27 ± 8.54%, while in *S. foetidus* types, the weight loss was 13.9 ± 3.25%, which was more stable than *I. oros*-derived membranes.

The weight loss of the SCFMs due to the collagenase enzymatic activity within 21 days is shown in Figure 7B. In both membrane types, the weight loss after seven days was not significantly different from the corresponding samples in PBS, resulting in 26.29 ± 5.25% and 18.03 ± 6.0%, respectively, while after 14 days in the enzyme treated cases the weight loss almost doubled. Specifically, in *I. oros*-derived membranes, a 58.93 ± 23.34% weight loss was detected, while in *S. foetidus*-derived membranes, it was 28.98 ± 0.06%. No further increase in weight loss was observed after 21 days in both samples. 

The evaluation of the organic compound release in saline or in collagenase solution from SCFMs during the in vitro degradation evaluation, obtained by the 280 nm absorbance in the membrane incubation media, has shown a similar trend in SCFM degradation rate (Figure 7C,D). Compared to the previous methods, here in the presence of collagenase, significant differences with respect to the PBS were detected already after 7 days.

To evaluate the percentage of collagen released or hydrolysed from the SCFMs within 21 days in PBS or in collagenase solution, the incubation media of the in vitro degradation test was hydrolysed and the hydroxyproline content was measured. When the membranes were incubated in PBS, no detectable hydroxyproline (<0.6 mg/mL) was found in each type of membrane. In the presence of collagenase in *I. oros*-derived membranes, the level of hydroxyproline recorded in the incubation media appeared to increase over time, resulting in 6.03 ± 0.89 μg/mL after 21 days, while in *S. foetidus*-derived membranes, the level of hydroxyproline was appreciable only after 21 days, resulting in 2.30 ± 0.37 μg/mL. (Table 4). These data indicate that the membrane weight loss observed in PBS incubation was not due to collagen release, but to different organic compounds, while when the membranes were incubated in collagenase solution, considering a presence of hydroxyproline on average of 6.5% (Table 1) in the collagen, only a 26.2% and a 18.5% of the weight loss was collagen in *I. oros*-derived membranes and *S. foetidus*-derived membranes, respectively. 

The greater stability of the *S. foetidus*-derived membranes compared to those derived from *I. oros*, evaluated in PBS and collagenase, obtained through the dry weight difference and through the analysis of the absorption at 280 nm of the incubation media could be due to the different textures of these membranes. These results are congruent with what was previously highlighted in relation to mechanical properties. The larger pore sizes in *I. oros*-derived membranes and the discontinuity of the intercellular collagen matrix that binds the filaments make this structure more susceptible to the dispersion of some of its components in water, and more accessible to collagenase digestion. Although it has previously been shown that sponge collagen is extremely resistant to collagenase treatment [33], the membranes in this study were subjected to greater weight loss after 14 days and a higher organic compound release after 7 days, compared to the saline solution, attesting a possible enzymatic activity. However, the analysis of the hydroxyproline level in the hydrolised membrane incubation media revealed that only a low percentage of weight loss was collagen material, suggesting an intimate association between collagen fibres and other organic materials that are solubilised together to the hydrolysed collagen. Moreover, the increased resistance to collagenase digestion exhibited by *S. foetidus*-derived membranes compared to *I. oros*-derived membranes could be probably linked to the partial coating of the filaments with the biomineral material, which could limit access to the enzymes. 

#### 2.2.5. Swelling Test and Antioxidant Activity 

The degree of swelling of SCFMs after immersion in water over a 24 h period is shown in Table 5. *I. oros*-derived membranes showed a percentage of swelling that was about double as compared to *S. foetidus* membranes (1511.44 ± 149.23% and 827.94 ± 64.40%, respectively). An extended immersion time of the membranes by an additional 24 h did not increase their weight (data not shown), showing that they had already reached their maximum swelling level after 24 h. The great difference in hydrophilicity between the two membrane types could be related to the abundant presence of iron biominerals in the surface of *S. foetidus* filaments. The addition of iron nanoparticles to collagen-derived biomaterial can in fact reduce its water affinity [42]. This inorganic component almost certainly increases the stability of the membranes in aqueous solutions, but at the same time, it reduces the surface interaction between water and the collagen fibres, and this definitively could decrease the membrane’s ability to bind water. The swelling behaviour of a material is driven not only by its chemical nature but also by its structure. Compared to membranes with the same surface area obtained by collagen fibres isolated from the marine sponge *C. reniformis* [11], the membranes described in this study showed higher hydration levels. Here, the greater dimensions of the collagen’s diameters increase the membrane thickness, thus improving the surface contact with water.

The above described membrane features strongly suggest some uses in regenerative medicine, in particular in tissue repair approaches. In the animal kingdom, including in mammalians and human beings, wound-healing processes are mostly accompanied by a local inflammatory response, generating reactive oxygen species and cell oxidative stress [43]. Thus, biomaterials designed for these applications are often conjugated with antioxidant compounds [44]. Marine sponges are known to be rich in secondary metabolites [45], many of which have antioxidant properties [46]; furthermore, sponge collagenic peptides seem to have, in certain instances, antioxidant properties [37]. Here, we have evaluated the radical scavenging activity of SCFMs using the DPPH assay. The results reported in Table 5 indicate that no significant antioxidant activity was detected in *I. oros*-derived membranes, while each 25 × 28 mm *S. foetidus*-derived membrane exhibited 57.24 ± 8.58% of antioxidant activity. Since both membranes are composed of collagen, in this specific case we cannot attribute a direct role of sponge collagen, but we suggest that the remarkable difference in antioxidant properties can be due to the presence of iron-based biominerals. This assumption can also be supported by the literature evidence, where the antioxidant properties of iron oxide nanoparticles of biogenic origin are extensively documented with proposed uses in biomedicine and bioremediation [47].

### 2.3. SCFM Biocompatibility Evaluation 

#### 2.3.1. Cell Adhesion and Cell Proliferation 

**Figure 8:**
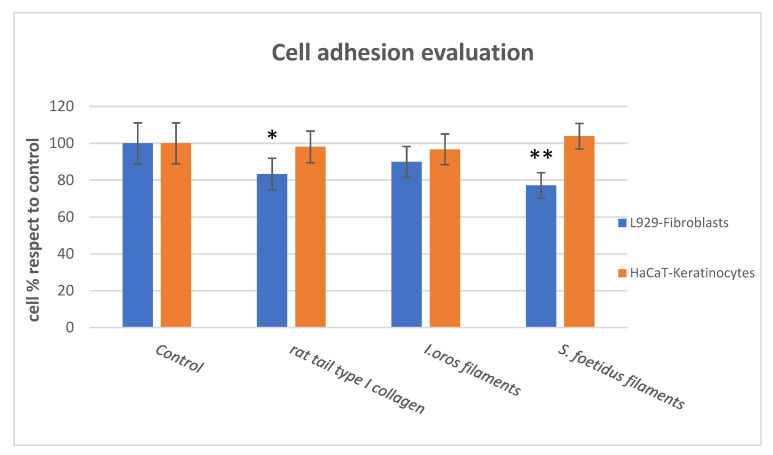
Cell adhesion evaluation. Cell adhesion quantitative evaluation, by MTT test, of L929 fibroblasts (blue bars) and HaCaT keratinocytes (orange bars) on the rat tail type I collagen, *I. oros* collagen filaments and *S. foetidus* collagen filaments pre-coated plates after 16 h of incubation. Results are expressed as cell percentages with respect to controls that were seeded on uncoated wells and are the mean ± S.D. of three experiments that were performed using eight well for each experimental condition. Statistical analysis results: one-way ANOVA, (blue bars) *p* < 0.05; (orange bars) *p* < 0.05. Asterisks indicate a significant difference versus the respective control (paired Tukey test, * *p* < 0.05, ** *p* < 0.001).

To evaluate the biocompatibility of SCFMs, L929 fibroblasts and HaCaT keratinocytes were grown on SCF-coated plates. Subsequently, a cell adhesion rate of 16 h after plating, and cell viability at three and six days were tested using MTT assay. Rat tail collagen was used as a comparison. No significant differences in cell adhesion were observed for both cell lines used on plates coated with *I. oros* collagenous filaments, compared to the uncoated plate controls after 16 h (Figure 8). Some reduced attachment of L929 fibroblast was observed on plates coated with *S. foetidus* collagen filaments, (77.14 ± 6.94%, see Figure 8), while no significant differences compared to the control were detected for HaCaT keratinocytes. An analogous slight reduction in L929 adhesion can be observed in the case of rat tail collagen coating used as a comparison (83.28 ± 8.57% of L929 fibroblasts were attached to the plates), while no significant differences compared to the control, were detected for HaCaT keratinocytes. Overall, these data showed that except for L929 fibroblasts plated on *S. foetidus* collagen filaments, no significant differences in cell adhesion rate were observed after 16 h in the other cases. 

**Figure 9:**
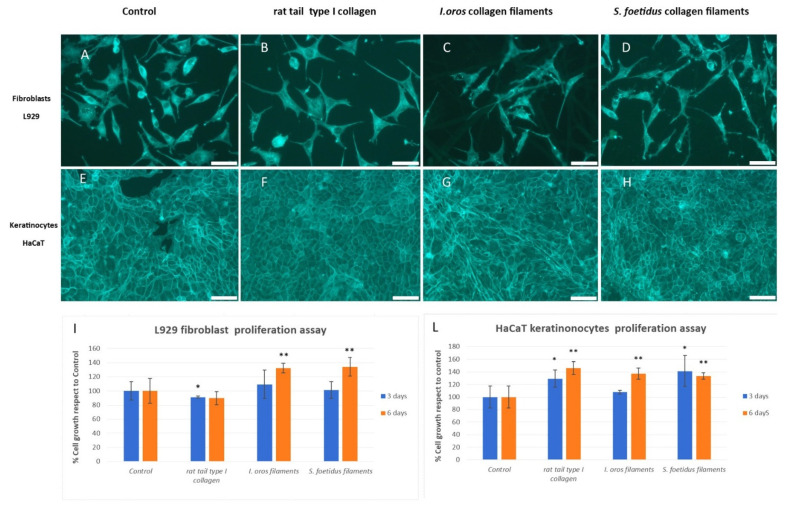
Cell proliferation evaluation. (**A**–**D**) Alexa Fluor 488-conjugated phalloindin stained L929 fibroblasts, epifluorescence microscope. (**A**) In control cells, the fluorescent actin cytoskeleton allows the observation of the typical aspect of fibroblast in culture, with fusiform, elongate shape, some focal adhesion (bright dots) and no preferential direction of cell and processes elongation. (**B**) Cells with rat tail type I collagen show no shape alteration. (**C**) *I. oros* collagen filaments are visible as weakly fluorescent stripes in the background. The fibroblasts clearly interact with the collagen and maintain their overall shape. (**D**) The thin collagen filaments of *S. foetidus* are not easily visible through the fluorescent filters; fibroblast have a morphology similar to the control. (**E**, **F**) Alexa Fluor 488-conjugated phalloindin stained HaCAT keratinocytes, epifluorescence microscope. (**E**) In control cells, the fluorescent actin cytoskeleton allows the observation of the organisation of keratinocytes that tend to interact, forming sheets. (**F**–**H**) The presence of collagen from rat tail, *I. oros* and *S. foetidus* does not induce detectable alterations in the morphology of cultured keratinocytes. Although covered by cells, the presence of collagen filaments of *I. oros* is detectable as two stripes from top left to the bottom right corner of the G photograph. Scale bars 50 µm. (**I**–**L**) Cell viability quantitative evaluation, by MTT test, of L929 fibroblasts (panel **I**) and HaCaT keratinocytes (panel **L**) on the rat tail type I collagen, *I. oros* collagen filament and *S. foetidus* collagen filament pre-coated plates after 3 days (blue bars) and 6 days (orange bars) of incubation. Results are expressed as cell percentages compared to controls that were seeded on uncoated wells, and are the mean ± S.D. of three experiments that were performed using eight well for each experimental condition. Statistical analysis results: one-way ANOVA, (I, blue bars) *p* < 0.0001, (I, orange bars) *p* < 0.0001; (L, blue bars) *p* < 0.0001, (L, orange bars) *p* < 0.005. Asterisks indicate a significant difference versus the respective control (paired Tukey test, * *p* < 0.05, ** *p* < 0.001).

**Figure 10:**
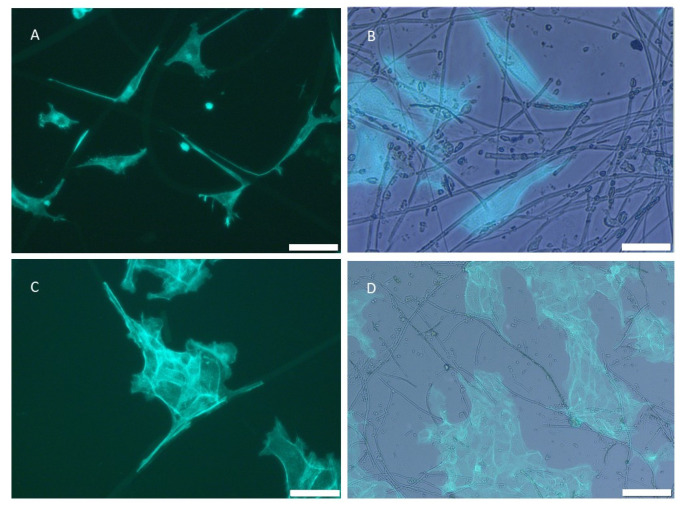
Alexa Fluor 488-conjugated phalloindin stained cell, epifluorescence microscope. (**A**) L929 fibroblasts and *I. oros* collagen filaments. Both the short and the long cell processes interact with the large collagen fibres that are visible because of a weak autofluorescence. The cell processes appear bended to adhere to the filaments. Scale bar 50 µm. (**B**) L929 fibroblasts and *S. foetidus* collagen filaments. As the thin filaments are not visible in the fluorescence photographs, the transmitted light image is overlapped to the fluorescence. Fibroblasts interact with collagen filaments. Scale bar 20 µm. (**C**) HaCAT keratinocytes and *I. oros* collagen filaments. The cells tend to form the usual sheets while interacting with the collagen filaments that are visible because of a weak autofluorescence. Scale bar 50 µm. (**D**) HaCAT keratinocytes and *S. foetidus* collagen filaments. As the thin filaments are not visible in the fluorescence photographs, the transmitted light image is overlapped to the fluorescence. The sheets of cells are in contact with the collagen filaments. Scale bar 100 µm.

Along with adhesion, in our experimental model, cell viability results are also reported here. The L929 fibroblast viability assay of SCFMs and rat tail collagen coating three and six days after plating is shown in Figure 9 (Panel I). Compared to the control, no significant differences were observed on plates coated with *I. oros* filaments after three days, while after six days, L929 fibroblasts growth resulted in 132.48 ± 6.83% compared to the control, attesting an improved proliferation rate. Similar results were observed in fibroblasts growing on plates coated with *S. foetidus* filaments. No difference in cell viability was observed with this biomaterial after three days, in comparison with the uncoated control sample, while after six days, the L929 fibroblast viability on this biomaterial was 134.19 ± 12.82% compared to the control. Conversely, on rat tail collagen coating, the cell viability after three days was 89.74 ± 9.40% compared to the control and no significant differences were registered after six days. Therefore, in our experimental conditions, in regards to the cell growth induction, SCF coating was more effective for fibroblast cell lines than mammalian collagen coating. Micrographs of the phalloidin-stained L929 fibroblasts growing on *I. oros* and *S. foetidus* collagen filaments, respectively, are shown in Figure 9A,B. After 24 h, their shape and their interaction with the biomaterials were very similar to the cell morphology observed in the controls and cells plated on rat tail collagen (Figure 9A,B, respectively). Figure 9 also shows the HaCaT keratinocytes proliferation assay (Panel L). Here, no significant differences in viability were detected on *I. oros* collagen filament coatings three days after plating, while after six days a significative increase in viability was observed (137.18 ± 8.97%), compared to the control. On *S. foetidus* collagen filament coatings, cell growth of 141.25 ± 24.61% and 133.33 ± 5.10% was registered after three and six days, respectively, compared to their controls. Similarly, in the rat tail collagen coating, a cell viability of 129.23 ± 13.85% and 146.15 ± 10.26% was measured after three and six days, respectively. Therefore, despite different temporal kinetics, SCF coatings are also able to promote in vitro cell proliferation of keratinocytes similar to that observed in mammalian collagen coatings. The phalloidin-stained HaCaT keratinocyte cells growing on *I. oros* and *S. foetidus* collagen filaments are shown in Figure 9G,H, respectively. As previously shown for fibroblasts, no significant morphology variations were observed compared to the control and rat tail coating samples (Figure 9E,F, respectively). Similar to what has been previously reported for spongin structures [48], the *I. oros* filaments emitted weak green autofluorescence, and this peculiar histological property, combined with their large size, allows a detailed observation of how both the fibroblasts and keratinocytes are intimately associated with the biomaterial and their prolongations (Figure 9C,G). In Figure 10, the way that the cell extensions faithfully follow the filaments’ shape can be observed in detail at a higher magnification (Figure 10A,C). Different to *I. oros*, *S. foetidus* filaments do not emit autofluorescence, but by superimposing the images obtained with fluorescence and light microscopy, it is still possible to observe that even with this biomaterial, both the fibroblasts and the keratinocytes are closely associated with the filaments, and their cell body often follows the filaments’ direction (Figure 10B,D). Together these data show us that regardless of their size or the presence of iron biomineral coverage, both sponge-derived collagen filaments exhibited good biocompatibility, and can improve fibroblast and keratinocytes proliferation. Furthermore, the direct cell surface interactions with these marine biomaterials and their ability to drive the cell growth direction along their structure are extremely suitable to guided tissue regeneration (GTR) applications [49].

#### 2.3.2. Fibroblast Gene Expression Analysis and Collagen Expression Level 

**Figure 11:**
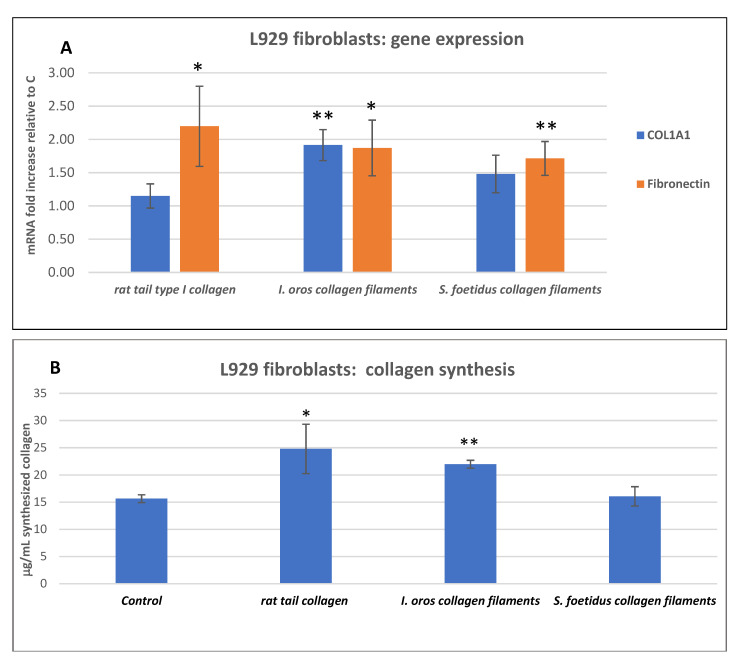
Gene expression profile of ECM production-related genes and fibrillar collagen expression level analysis in L929 fibroblast growing on SCMs. (**A**) qPCR evaluation of type I collagen (COL1A1) and fibronectin gene expression (blue and orange bars, respectively) in L929 mouse fibroblasts growing 24 h on wells precoated with rat tail type I collagen, *I. oros* collagen filaments and *S. foetidus* collagen filaments. Data are expressed as fold increase relative to the control (uncoated wells) and normalised to GAPDH gene. Each bar represents the mean ± S.D. of two independent experiments performed in triplicate. (**B**) Fibrillar collagen synthesis quantification measured using the Sircol colorimetric method as described in Section 4.8.4 of L929 fibroblast growing 48 h on uncoated wells (control) or precoated with rat tail type I collagen, *I. oros* collagen filaments and *S. foetidus* collagen filaments. Statistical analysis results: one-way ANOVA, (**A**) *p* < 0.005; (**B**) *p* < 0.001. Asterisks indicate a significant difference versus the respective control (paired Tukey test, * *p* < 0.05, ** *p* < 0.001).

In the early phases after injury, various extracellular matrix proteins have been released during dermal reconstitution in the wound-healing process [50,51]. To assess whether SCFMs can induce ECM production-related gene up-regulation, α1 chain of collagen type I (COL1A1) and the fibronectin gene expression profile were evaluated by qPCR in L929 fibroblasts growing on plates coated with *I. oros* and *S. foetidus* collagen filaments. Rat tail collagen coating was used as a comparison. As shown in Figure 11 (Panel A), a COL1A1 and fibronectin gene expression increase of 1.91 ± 0.23 and 1.87 ± 0.45 folds, respectively, was observed in cells plated on *I. oros* filaments after 24 h compared with the control. In cells plated on *S. foetidus* filaments, no significant COL1A1 mRNA level fold increase was detected compared to the control, while a fibronectin mRNA fold increase of 1.7 ± 1.25 was registered, as has been observed when fibroblasts were grown on rat tail collagen coating, where no significant COL1A1 gene up-regulation was detected, while fibronectin mRNA was 2.2 ± 0.6 fold higher than the control sample. The positive effect on collagen production generated by the interaction of L929 fibroblasts with *I. oros* collagen filaments was further confirmed by the biochemical evaluation of the tropo-collagen expression level in the culture medium within 48 h after plating. In our experimental conditions, collagen production was significantly increased by 40% in fibroblasts growing on this biomaterial compared to the control sample, as shown in Figure 11 (Panel B). This increment of collagen secretion appears not significantly different from what was observed in fibroblasts growth on rat tail collagen coating. However, in the L929 plated on *I. oros* filaments, the increase in collagen production is clearly dependent on an up-regulation of COL1A1 gene expression level, while a different mechanism seems to be activated in the case of L929 plated on rat tail collagen, among which a possible contribution from the degradation action of the coating on which the fibroblasts are plated cannot be excluded, where no significant COL1A1 mRNA increase was observed. (Figure 11, panel A, blue bar). To date, the induction of collagen biosynthesis by sponge-derived collagen has only been reported when it is enzymatically hydrolysed into short bioactive peptides [37]. This study for the first time demonstrates a direct effect of intact collagen filaments used as growth scaffolds on the collagen expression level in in vitro fibroblasts. Microscopic analysis did not reveal evident differences with respect to controls, suggesting a possible differentiation to myofibroblasts of L929 cells plated on SCFMs, however further molecular studies may better elucidate the pathway involved in the up-regulation of collagen and fibronectin. However, it is known that fibroblasts can respond to mechanical forces by altering their expression of a specific gene or proteins involved in differentiation and growth [52]. The different response to the collagen gene expression of L929 cell line in the two types of SCFMs could be related to the different porosity of these biomaterials, inducing differences to mechanical loading.

## 3. Conclusions

In this study, the biomedicine applicative potential of unique collagen structures extracted from keratose sponges belonging to the genera *Ircinia* and *Sarcotragus* has been evaluated for the first time. Their peculiarity derives from an interesting organisation of sponge collagen characterised by high species specificity, where shape and size of the filaments strongly vary in the animal species. Here, we have verified the potential use of two very different types of filaments, derived from *I. oros* and *S. foetidus*, to produce 2D membranes for cell and tissue culture. The data obtained showed that these marine biomaterials, once purified, could be particularly suitable to produce 2D films for wound-dressing applications, as they combine mechanical strength, stability in saline solutions, antioxidant properties and biocompatibility. Particularly, *I. oros*-derived membranes, compared with those derived from *S. foetidus*, showed higher thermal stability and swelling properties. Conversely, *S. foetidus*-derived membranes exhibited higher mechanical resistance, good stability in saline and collagenase solutions and antioxidant properties. Both membrane types can promote cell growth and fibronectin gene up-regulation in fibroblast cells line. *I. oros*-derived membranes could also stimulate a strong collagen production. The peculiarities of these two different marine biomaterials highlighted in this study further confirm the extraordinary applicative potential of these marine sponges in the innovative biomaterials field. The development and optimisation of effective marine aquaculture systems combined with that of the filament extraction processes would allow the large-scale creation of new materials, inspired by nature, for extremely high-performing biomedical use.

## 4. Materials and Methods

### 4.1. Chemicals

All reagents were acquired from SIGMA-ALDRICH (Milan, Italy), unless otherwise stated.

### 4.2. Sponge Sampling

Specimens of *Ircinia oros* (Schmidt, 1864) and *Sarcotragus foetidus* (Schmidt, 1862) were harvested from scuba diving in the area of the Portofino Promontory (Liguria, Italy) at depths of 10–20 m, and were transferred to a laboratory in a thermic bag maintained at 14–15 °C. The sponge specimens were frozen at −20 °C until further processing.

### 4.3. Sponge Collagenous Filaments and Intercellular Collagen Isolation

**Scheme 1:**
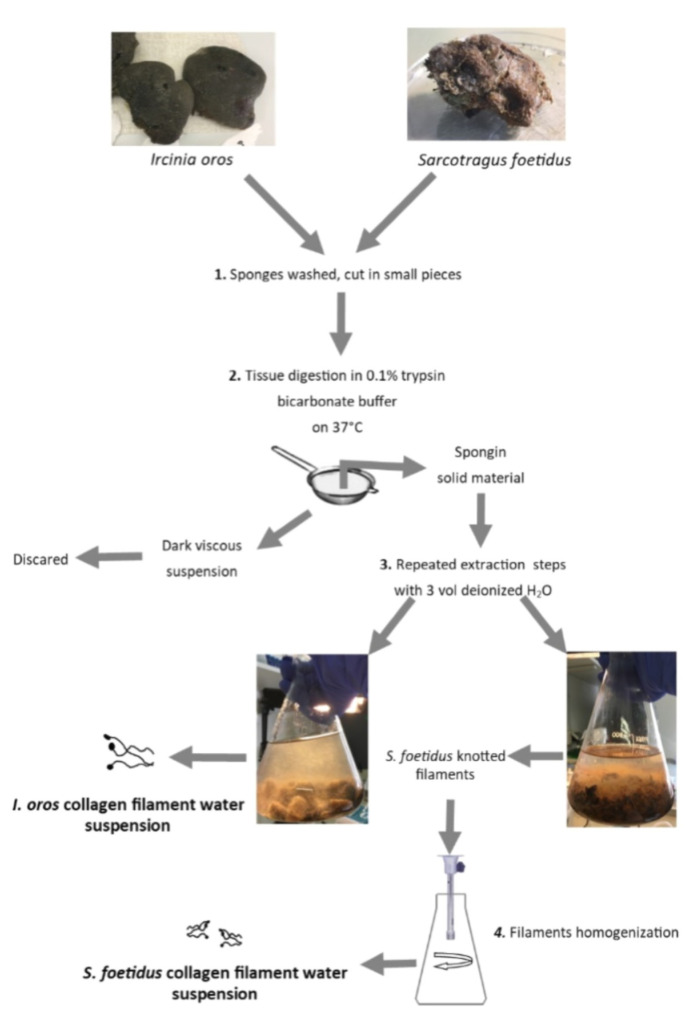
Schematic representation of the extraction procedure used to isolate the collagen filaments from *I. oros* and *S. foetidus* tissue.

Frozen sponges were extensively washed in running tap water to remove sand residues, and finally washed with distilled water. The sponge tissues were minced in small pieces with scissors and enzymatically digested, as previously described [18]. The flowsheet of the extraction procedure is shown in Scheme 1. A total of 20 g of cut sponge tissues were treated with 0.1% trypsin in 100 mL of ammonium bicarbonate, pH 8.5, overnight at 37 °C on a horizontal shaker. Subsequently, the dark fluid was removed by filtration with a metallic strainer, and the solid material was suspended in three volumes of cool deionised water and incubated at 5 °C for three days in a rotary disc shaker aliquoted in 50 mL tubes. 

This treatment efficiently disperses the intercellular collagen fibrils in water, leaving only the brown spongin scaffold combined with the collagenous filaments in the final residue. These last structures, combined with residual intercellular collagen, were finally separated from the fibrous spongin matrix through different rounds of sedimentation steps under gravity, by mild stirring in large volumes of distilled water and repeated decantations. To obtain a homogenous suspension for *S. foetidus* collagenous filaments, the sample was subjected to a short round of homogenisation steps in ice for 15 s at the end of the extraction using Ultra Turrax T25 basic (IKA-WERKE, Staufen im Breisgau, Germany). During all the extraction phases, the filaments’ suspension was never subjected to centrifugation steps to avoid knotting the filaments. The filaments were extensively washed with distilled water to allow them to settle by gravity. The extracted sponge collagenous filament suspensions were conserved at 4 °C. To establish the concentration of the sponge collagen filaments, 1 mL of each suspension was lyophilised, and the dry material was weighed.

### 4.4. Light Microscopy and Environmental Scanning Electron Microscope (ESEM) Observation

*I. oros* and *S. foetidus* tissue fragments, isolated sponge collagenous filaments and sponge collagenous filament membranes (SCFMs) were observed in light microscopy through a stereomicroscope (Nikon SMZ1000, Nikon, Tokyo, Japan) equipped with a digital camera (digital Sight DS-SM, Nikon). Isolated sponge collagenous filaments were coloured with picro-sirius red, as described in [53], in order to prove their collagen composition. For ESEM observation, isolated sponge collagenous filaments and SCFMs were firstly completely dehydrated by soaking them in a series of alcoholic solutions with an increasing concentration of ethanol up to 100%, then graphite was covered and examined. Images of the samples were observed and acquired with an ESEM Vega3–Tescan, type LMU (Tescan Brno s.r.o., Brno, Czech Republic) provided with a microanalyser system EDS-Apollo_x and EDS texture and elemental analytical microscopy software (TEAM^TM^ Analysis System, version 1.0, Coherent Scientific, Thebarton SA). Observation and acquisition of the four SCFMs images were performed with a FESEM Zeiss SUPRA 40 VP (Carl Zeiss AG, Oberkochen, Germany) and its associated software. The fibrillar diameter and the pore areas observed in the collagen membranes were analysed by performing physical measurements on the images of the various membranes acquired with the FESEM, using the ImageJ free software (version 1.53 Rasband, W.S., ImageJ, U.S. National Institutes of Health, Bethesda, MD, USA, https://imagej.nih.gov/ij/, 1997–2016). Means ± S.D. were calculated on at least 40 random measurements of fibril diameter or pore areas performed on each membrane.

### 4.5. SCFs Biochemical Characterisation

#### 4.5.1. Amino Acid Composition

Amino acid analysis was performed using a Jasco X- LC system equipped with an autosampler, Xtreme high pressure pumps, degasser, column oven compartment, fluorescence detector connected to a HP ProDesk processor, as described in [54]. In total, 0.5 mL of each sponge collagenous filament suspensions at 2 mg/mL were hydrolysed in NaOH 2 N for 20 min a 121 °C at 1 Atm and neutralised with equal volume of HCl 2N.

Hydrolysed amino acids were then derivatised by ortho-phthalaldehyde (OPA) and fluorenylmethylchloroformate (FMOC), leading to the formation of derivatives from primary amino acids and secondary amino acids, respectively. Derivatisation was performed accordingly wiyh Jasco autosampler program. The derivatives were detected with a fluorometric detector (emission λ = 446 nm – excitation λ = 340 nm for OPA derivatives and emission λ = 268 nm – excitation λ = 308 nm for FMOC derivatives). Amino acid identification was performed via elution times of the obtained derivatives and compared to a mixture of standard amino acids submitted to derivation in identical test conditions. Data processing software (ChromNav, version 2.0, JASCO, Inc., Easton, MD, USA) allowed integration of peak areas for the assessment of the amount of amino acids occurring in the sample. 

#### 4.5.2. Glycosaminoglycans (GAGs) Quantification

The quantitative evaluation of the GAGs content in sponge collagen filaments was obtained using the Alcian blue assay as described in [11] and expressed as μg of dry weight of collagen filaments.

#### 4.5.3. Quantitative Analysis of Iron Content 

The μg of iron present in sponge collagen filaments was obtained by inductively coupled plasma atomic emission (ICP–AES), as described in [55].

#### 4.5.4. Sodium Dodecyl Sulfate Poly Acrylamide Gel Electrophoresis (SDS-PAGE) 

Protein patterns of sponge collagenous filament samples were analysed by SDS–PAGE using Mini-Protean 3 (Bio-Rad Laboratories, Hercules, CA, USA), according to the method previously described [56]. A total of 0.5 mL of each sponge collagen filament suspension normalised at 4 mg/mL were added to an equal to volume of acid-washed glass beads (0.5 mm diameter) and vortexed 3 times for 30 s. Samples were mixed at 1:1 (*v/v*) ratio with 2× gel loading buffer (1 M Tris–HCl buffer (pH 6.8), 10% 2-mercaptoethanol, 40% glycerol, 0.2% bromophenol blue and 20% Sodium Dodecyl Sulfate solution) and was heated at 90 °C for 5 min, and 40 μL were loaded in a 7% of polyacrylamide gel and run at 60 mA with constant amperage. After electrophoresis, the gel was fixed for 1 h a room temperature in a solution containing 10% (*v/v*) acetic acid and 40% (*v/v*) ethanol), washed twice for 10 min at room temperature with distilled water and stained over night at room temperature with a staining solution obtained combing 80 mL of colloidal Coomassie solution (0.1% (*w/v*) Coomassie Brilant blue G250, 2% (*w/v*) ortho-phosphoric acid, 10 (*w/v*) ammonium sulphate) with 20 mL of methanol. Finally, the gel was destained with 5% acetic acid solution and acquired with ChemiDoc Imaging System (Bio-Rad, Milan, Italy). Rat tail type I collagen (0.5 mg/mL) was run alongside as control. 

### 4.6. SCFMs Production

SCFMs were obtained by casting 2 mg/mL of sponge collagenous filament suspension in silicone moulds as rectangular (25 × 28 mm) sheets that were filled with 3.3 mL of 2 mg/mL of each suspension, and as rectangular (10 × 45 mm) sheets for mechanical tests, filled with 2.25 mL of 2 mg/mL of each SCF suspension and let completely dry at 37 °C overnight. For DSC analysis, 3 mg of sponge collagenous filaments were left to dry directly on metallic melting pot. For biocompatibility tests, 2 mg/mL of sponge collagenous filament suspension derived from each sponge species and a standard rat tail collagen were used to directly coat 24-well and 96-well plates. In total, 300 μL (for 24-well plates) or 50 μL (for 96-well plates) were placed on the plates and were left to dry at 37 °C overnight. The coated plates were then washed thrice with 100% ethanol, UV sterilised for 20 min and conserved at 4 °C until use. 

### 4.7. SCFMs Characterisation 

#### 4.7.1. Differential Scanning Calorimetry 

DSC was performed using a DSC1 STAR^e^ System (Mettler-Toledo, Switzerland). About 3 mg dry SCFs were placed in aluminium crucibles and analysed with increasing heat from 0 to 200 °C at a heating rate of 5 °C/min. During the DSC runs, a nitrogen flow at a rate of 20 mL/min was constantly applied. 

As a control, 3 mg of a commercial porcine collagen membrane Bio-Gide® (Geistlich Pharma AG, Wolhusen, Switzerland) was dried on a metallic melting pot and analysed on the DSC following the same procedure outlined above.

#### 4.7.2. DMA|DMTA

Dynamic mechanical (DMA) and dynamic mechanical-thermal analysis (DMTA) were carried out on the SCFMs via an MCR 301 rheometer (Anton Paar, GmbH, Graz, Austria) equipped with a universal extensional fixture (UXF) geometry and a CDT-450 chamber. Rectangular specimens (40 mm × 10 mm) were prepared from the samples by using a punch cutter, and each sample thickness was measured via a digital micrometre. A static extensional stress (σ_s_) of 2 MPa was applied for all experiments to ensure the correct sample loading and result reliability.

The linear viscoelastic region (LVER) of the samples was first explored via amplitude sweep tests (AS) at T = 25 ± 1 °C using a frequency (ν) and oscillatory extensional stress (σ) of 1 Hz and in the range 0.01–10%, respectively. Then, frequency sweep tests (FS) were carried out at T = 25 ± 1 °C with a fixed σ = 0.1 MPa, varying the frequency between 0.01 and 10 Hz. Finally, temperature sweep tests (TS) were carried out in the temperature range 25–100 °C with a heating rate of 2 °C/min.

#### 4.7.3. In Vitro Degradation Study

To measure the in vitro degradation, each membrane type was weighed (designated as “Wi”) and transferred in a test tube containing 10 mL of PBS (pH = 7.4) alone or added with 0.1 mg/mL collagenase from *Clostridium histolyticum* and kept at 37 °C. After 7, 14 and 21 days, the samples were recovered, washed twice with deionised water, dried and weighed again (designated as “Wf”). The percent degradation of the membranes was computed by the following equation:

percent degradation (%) = (Wi − Wf) / Wi × 100%

The procedure was carried in triplicate.

To evaluate the release of organic compounds from the SCFMs in saline or collagenase solution withing 21 days, the absorbance at 280 nm was read in the membrane incubation media after 7, 14 and 21 days using PBS or collagenase solution as a blank sample, respectively. 

To detect the percentage of collagen material released in saline solution or hydrolysed by collagenase activity from SCFMs during the stability test, 0.4 mL of the incubation media was recovered at 7, 14 and 21 days, hydrolysed in NaOH 2 M at 120 °C at 1 Atm, and finally hydroxyproline content was evaluated as previously described [11].

#### 4.7.4. Swelling Test

To evaluate the water binding property of SCFMs, the initial weight of dried membrane (Wi) was measured and then they were soaked in deionised water at 37 °C for 20 h. Finally, the wet weight of the scaffolds (designated as “Ww”) was again recorded. A second weighing of the samples after 48 h of incubation without registering a further weight gain ensured that the samples had reached their maximum degree of hydration. Finally, the water content was calculated based on the equation:

water content (%) = (Ww − Wi) / Ww × 100%

Experiments were performed in triplicate.

#### 4.7.5. DPPH Radical Scavenging Activity

The radical scavenging activity was evaluated on each type of SCFMs as described in [11]. Briefly, 25 × 28 mm membranes were soaked into 500 μL of deionised water, and then embedded into 250 mL of 0.1 mM DPPH in methanol solution (2,2-diphenyl-1-picrylhydrazyl, Calbiochem®, Millipore SpA, Milan, Italy). In the same manner, a negative control sample with deionised water was prepared. The samples were left in incubation for 30 min at room temperature in the dark. Then, the membranes were removed with tweezers, and finally the sample solutions were read at 517 nm using a Beckman spectrophotometer (DU 640). The blank sample was prepared by replacing the DPPH solution with methanol. The antioxidant activity of the samples was evaluated by the inhibition percentage of DPPH radical using the following equation:

DPPH radical scavenging activity (%) = (A0 − A)/A0 × 100%

where A was sample absorbance rate; A0 was the absorbance of the negative control. The procedure was carried out in triplicate.

### 4.8. SCM Biocompatibility Evaluation

#### 4.8.1. Cell Cultures

The L929 mouse fibroblast cell line was obtained by the National Collection of Type Cultures (NCTC), while the human keratinocyte HaCaT cell line (CLS Cell Lines Service, 300493) was obtained by the Cell Lines Service (GmbH, Eppelheim, Germany).

Cells were maintained at 37 °C in a humidified, 5% CO_2_ atmosphere, in high glucose Dulbecco’s modified Eagle’s medium (D-MEM) with glutamax (Euroclone, Milan, Italy), which was supplemented with 10% FBS (Euroclone) and with the addition of penicillin/streptomycin as antibiotics.

#### 4.8.2. Cell Growth and Cell Adhesion

L929 and HaCaT cell lines were seeded at a density of 50,000 cells/well on 96-well plates that were, or not, pre-coated with each type of SCFs, or rat tail standard collagen as described in Section 4.4 for evaluating cell adhesion. Cells were allowed to adhere for 16 h at 37 °C in complete medium; the medium was subsequently removed, the adhered cells were washed once with PBS to remove the floating unattached ones, and finally MTT (0.5 mg/mL final concentration) test was performed as well to estimate the amount of attached cells when compared to control cells on uncoated wells. Data are means ± S.D. of four independent experiments.

To evaluate cell growth on SCF-coated plates, experiments were performed on 96-well plates. L929 and HaCaT cell lines were both plated at a density of 5000 cells/well on, or not, pre-coated wells. Cells were cultured for 3 days and 6 days at 37 °C in complete medium. At the end of the experiments, the MTT test was once again performed to evaluate cell viability.

For image acquisition, light microscopy cells were seeded at a density of 200,000 cells/well on 12-well plates pre-coated or not, with SCF or rat tail collagen and incubated for 24 h at 37 °C. At the end of the experiment, cells were washed once with PBS, fixed for 30 min at room temperature with 4% paraformaldehyde and then dehydrated in a 70% ethanolic solution. After fixation in buffered 4% paraformaldehyde, cells were rinsed for 5 min three times in PBS (0.1 M, pH 7.4), permeabilised for 10 min in 0.2% Triton-X 100 in PBS and thus rinsed again. Then, they were incubated in a blocking solution with 1% bovine serum albumin and 0.1% Tween 20 in PBS. After rinsing, cells were incubated in a moist dark chamber with Alexa Fluor 488-conjugated Phalloidin (1:40 in PBS, Invitrogen) for 30 min at room temperature. For image acquisition, an inverted optical microscope (IX53 Olympus, Tokyo, Japan) equipped with a CCD camera (U-LH100HG Olympus, Tokyo, Japan) was utilised, and the relative software was used.

#### 4.8.3. L929 Fibroblast Gene Expression Analysis 

L929 mouse fibroblast cells were seeded at a density of 200,000 cell/well on 12-well plates that were, or not, pre-coated with SCFs, or rat tail standard collagen, and were incubated for 24 h at 37 °C. At the end of the experiment, total RNA was extracted using the RNeasy Mini Kit, (Qiagen, Milan, Italy) according to the manufacturer’s instructions. 

The cDNA was synthesised by Revert Aid Reverse Transcriptase (Thermo Fisher Scientific, Milan, Italy) using 1 μg of purified total RNA from each sample. Each PCR reaction was performed in 15 μL containing: 1× master mix iQ SYBR®Green (Bio-Rad), 0.2 μM of each primer and 3 μL of a 1:5 diluted reverse transcription reaction buffer. Each sample was analysed in triplicate. The following thermal conditions were used: initial denaturation at 95 °C for 3 min, followed by 45 cycles with denaturation at 95 °C for 15 s and annealing and elongation at 60 °C for 60 s. At the end of each elongation step, fluorescence was measured. Values were normalised to GAPDH (reference gene) mRNA expression. All the PCR primers (Table S1) were designed by means of the Beacon Designer 7.0 software (Premier Biosoft International, Palo Alto, CA, USA) and obtained from TibMolBiol (Genova, Italy). Data analyses were acquired by the DNA Engine Opticon® 3 Real-Time Detection System Software program (3.03 version) and, in order to calculate the relative gene expression compared to an untreated (control) calibrator sample, the comparative threshold Ct method was used within the gene expression analysis for iCycler iQ Real Time Detection System Software® (2004 Bio-Rad, Milan, Italy). Data are means ± S.D. of two independent experiments performed in triplicate.

#### 4.8.4. L929 Fibroblast Collagen Synthesis Evaluation

Collagen synthesis by L929 fibroblasts was quantified in the cell medium by applying the SIRCOL^TM^ Soluble Collagen Assay (Biocolor Ltd., Carrickfergus, Northern Ireland, UK). Fibroblasts were seeded in tissue culture 12-well plates at a density of 200,000 cells/well that were, or not, pre-coated with each type of SCF and incubated for 48 h at 37 °C. 

At the end of the incubation, cell culture media were collected, and the SIRCOL assay was performed according to the manufacturer’s instructions. Data are the means ± S.D. of two independent experiments performed in triplicate. Cell culture media alone or pre-incubated for 48 h a 37 °C with the respective coating tested was used as a blank sample for control or for each sample, respectively. 

### 4.9. Statistical Analyses

Statistical analyses were performed using one-way ANOVA plus Tukey’s post test (GraphPad Software, Inc., San Diego, CA, USA). *p* values < 0.05 were considered to be significant.

## Figures and Tables

**Table 1:** Amino acid composition of *I. oros* and *S. foetidus* collagen filaments compared with collagen from skin of codfish and commercial rat collagen (per 1000 residues).

Amino Acid	*I.oros*	*S. foetidus*	Rat *	Codfish *
Ala+Arg	152	157	153.4	121.93
**Aspartic acid**	**100.03**	**78.83**	**45.32**	**38.82**
Glutamic acid	111.90	114.27	73.33	56.08
Glycine	359.00	385.46	333.18	266.12
Histidine	0.96	1.34	3.61	5.01
**Hydroxylysine**	**13.91**	**14.26**	**9.33**	**6.65**
**Hydroxyproline**	**69.29**	**61.53**	**96.09**	**39.6**
Isoleucine	8.57	5.62	7.48	5.61
Leucine	23.19	18.96	23.29	6.51
**Lysine**	**13.80**	**12.00**	**27.07**	**19.62**
Methionine	1.64	1.80	8.03	15.04
Phenylalanine	13.31	10.42	14.62	12.7
**Proline**	**57.06**	**43.56**	**109.21**	**62.69**
Serine	41.65	61.28	42.74	53.87
Threonine	18.53	18.66	18.79	16.89
Tyrosine	4.99	5.17	3.76	2.25
Valine	10.59	10.18	17.08	12.02

* Value obtained from [36].

**Table 2:** Comparison between *I.oros* collagen filaments and *S.foetidus* collagen filaments in amino acids, GAGs and iron content (μg/mg dry collagen filaments).

-	Amino Acids (μg/mg)	GAGs (μg/mg)	Iron (μg/mg)
***I. oros***	490.3 ± 3.4	12.28 ± 5.4	2.7 ± 0.32
***S. foetidus***	250.9 ± 2.9	28.49 ± 8.3	45.32 ± 2.8

**Table 3:** Mechanical test of SCFMs. DMA.

Sample	E’ @ 1 Hz, 25 °C (MPa)	St. Dev	E" @ 1 Hz, 25 °C (MPa)	St. Dev
*I. oros*	447.15	9.49	18.52	0.86
*S. foetidus*	819.27	37.88	44.25	4.50

**Table 4:** Hydroxyproline content the incubation media of the in the vitro degradation test.

Time (days)	*I. oros*	*S. foetidus*
	μg/mL	μg/mL
7	3.64 ± 0.56	<0.6
14	5.78 ± 0.28	<0.6
21	6.03 ± 0.89	2.30 ± 0.37

**Table 5:** Swelling index and antioxidant activity evaluation of SCFMs derived from *I. oros* and *S. foetidus.* (*n* = 3, mean ± standard deviation).

Sample	Swelling Index (%)	Antioxidant Activity (%)
*I. oros*	1511.44 ± 149.23	4.64 ± 1.78
*S. foetidus*	827.94 ± 64.40	57.24 ± 8.58

## Data Availability

Not applicable.

## Supplementary Materials

The following are available online at https://www.mdpi.com/article/10.3390/md19100563/s1, Figure S1: EDS spectra of *S. foetidus* filaments. Figure S2: *S. foetidus* collagen filament in native tissue. Table S1: S1 primer sequences used in the qPCR analyses.
