# Supplementary material for: Potential Biomedical Applications of Collagen Filaments derived from the Marine Demosponges Ircinia oros (Schmidt, 1864) and Sarcotragus foetidus (Schmidt, 1862)
Source: Mar Drugs. 2021 Oct 6;19(10):563. doi: 10.3390/md19100563 (PMC8540060; doi:10.3390/md19100563)
Supplement: Supplementary file 1 [file marinedrugs-19-00563-s001.zip › marinedrugs-1331956-supplementary.pdf]

Supplementary materials

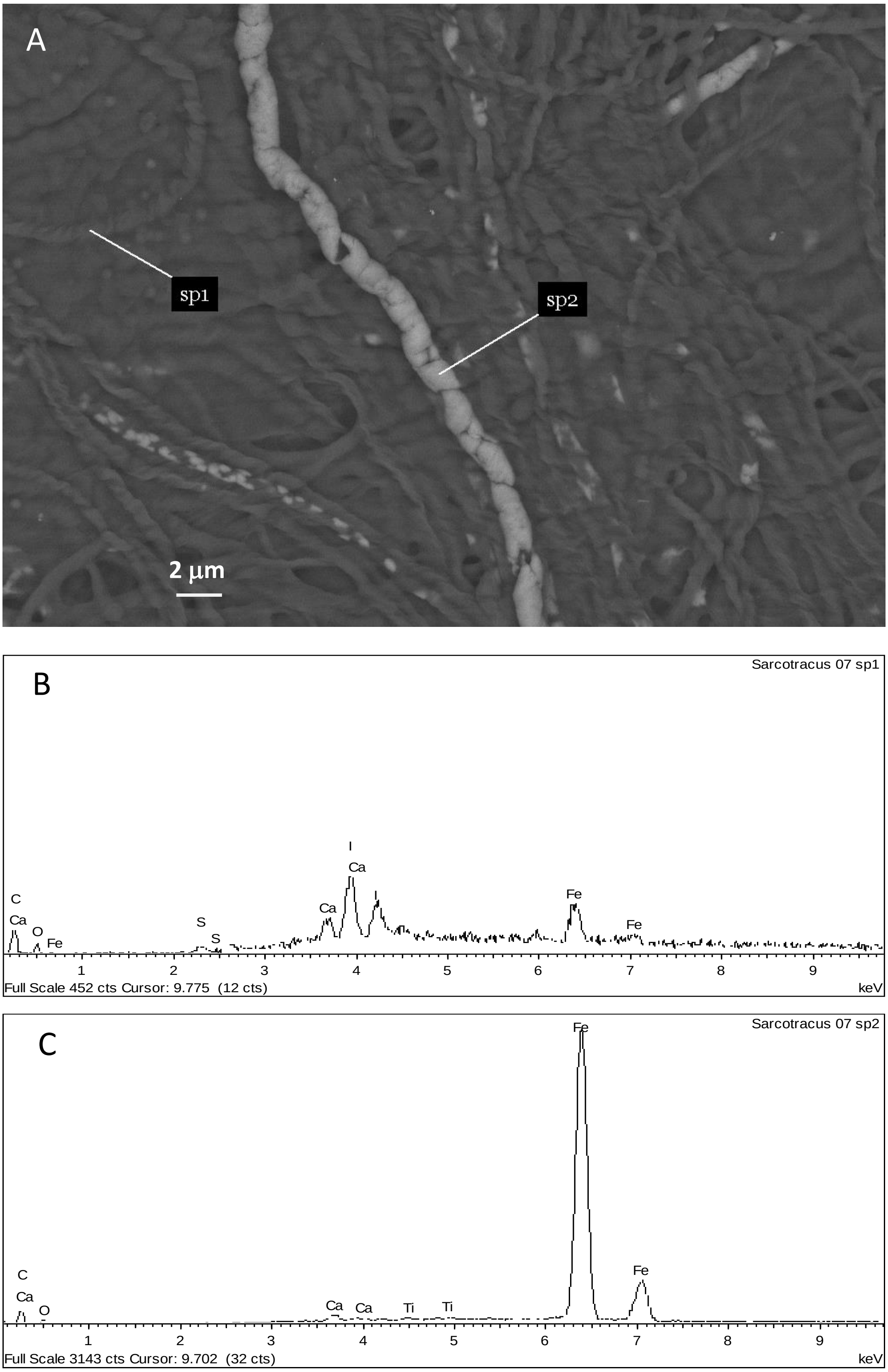

Figure S1 EDS spectra of *S. foetidus* filaments. Electronic microscopy images (A). EDS spectra sp1 (B). EDS spectra sp2 (C).

Figure S2

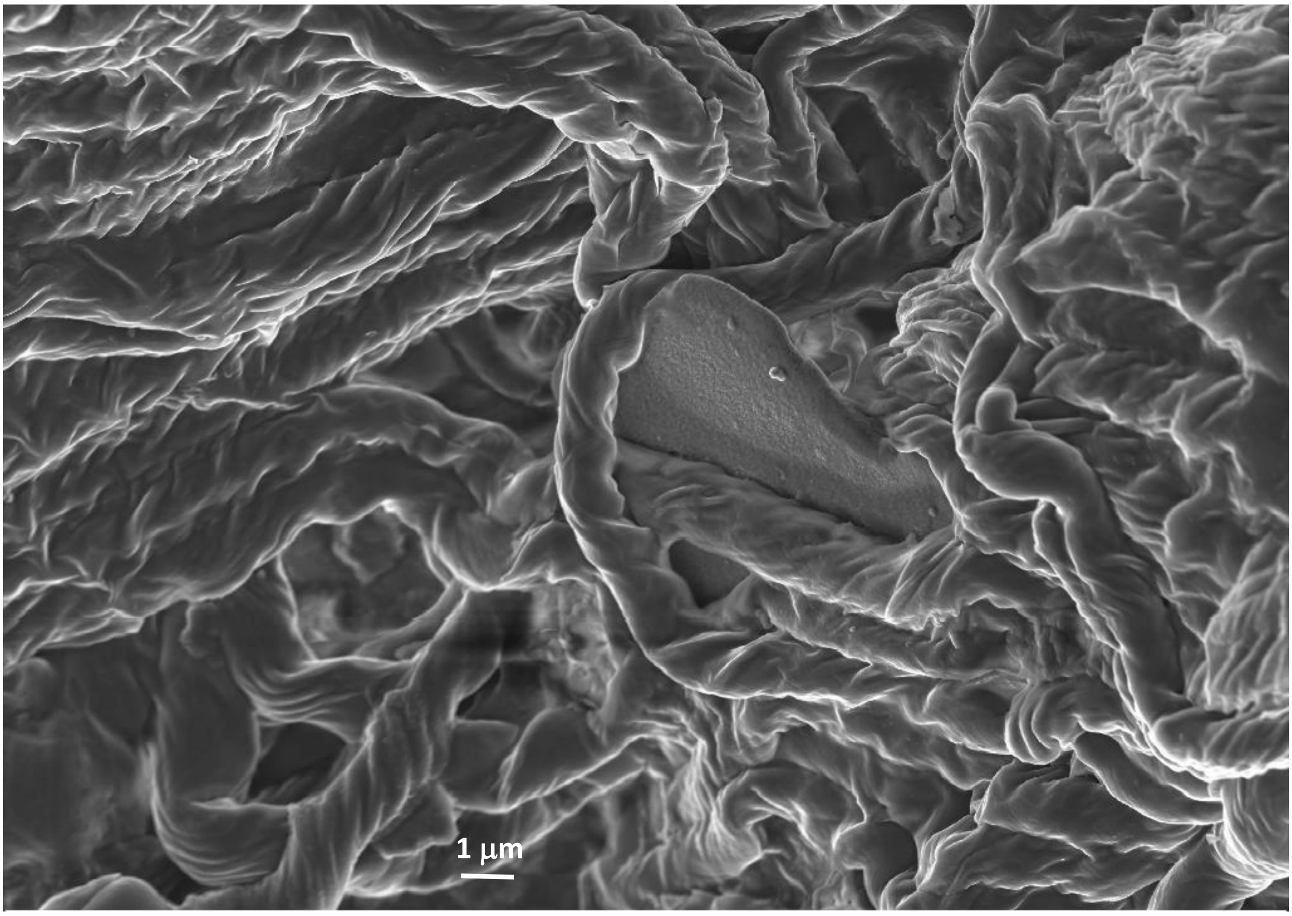

Figure S2. *S. foetidus c*ollagen filament in native tissue.

Table S1 Primer sequences used in the qPCR analyses.

Gene	GeneBank id	Forward	Reverse
GAPDH	M32599	5'-TCTCCCTCACAATTTCCATCCCAG-3'	5'-GGGTGCAGCGAACTTTATTGATGG-3'
Col1A	NM_007742.4	5'-CTGCTGGTCCTGCTGGTC-3'	5'-CCTTGTTCGCCTGTCTCAC-3'
Fn1	NM_001276412	5'-CCAGTTCAGAGGAGCATCAG-3'	5'-GGCATTGTCGTTCAGAGTGTA-3'
